# Curcumin inhibits leptin gene expression and secretion in breast cancer cells by estrogen receptors

**DOI:** 10.1186/1475-2867-14-66

**Published:** 2014-12-23

**Authors:** Kazem Nejati-Koshki, Abolfazl Akbarzadeh, Mohammad Pourhassan-Moghaddam

**Affiliations:** Department of Medical Biotechnology, Faculty of Advanced Medical Sciences, Tabriz University of Medical Sciences, Tabriz, Iran; Department of Medical Nanotechnology, Faculty of Advanced Medical Sciences, Tabriz University of Medical Sciences, Tabriz, Iran; Department of Clinical Biochemistry, Faculty of Medicine, Tabriz University of Medical Sciences, Tabriz, Iran

**Keywords:** Leptin, Curcumin, Breast cancer, T47D cell line

## Abstract

**Background:**

Recent studies suggested that leptin as a mitogenic factor might play an important role in the process of initiation and progression of human cancer. Therefore, it could be considered as a target for breast cancer therapy. A previous study has showed that expression of leptin gene could be modulated by activation of estrogen receptors. Curcumin is a diferuloylmethane that has been shown to interfere with multiple cell signaling pathways and extensive research over the last 50 years has indicated this polyphenol can both prevent and treat cancer. Based on the fact that targeting of leptin could be considered as a novel strategy for breast cancer therapy, the aim of this study is the investigation of potentiality of curcumin for inhibition of leptin gene expression and secretion, and also, its link with expression of estrogen receptors.

**Methods:**

Cytotoxic effect of curcumin on T47D breast cancer cells was investigated by MTT assay test after 24 and 48 treatments. Thereafter, the cells treated with different concentrations of curcumin. The levels of leptin, estrogen receptor α and estrogen receptor β genes expression was measured in the treated and control cells by Reverse-transcription real-time PCR. Amount of secreted leptin in the culture medium was also determined by ELISA in both treated and untreated cells. Finally data were statistically analyzed by one-way ANOVA test.

**Results:**

Analysis of MTT assay data showed that curcumin inhibits growth of T47D cells with dose dependent manner. There were also significant difference between control and treated cells in the levels of leptin, estrogen receptor α expression levels and the quantity of secreted leptin that both were decreased in the treated cells compared with control cells.

**Conclusion:**

Based on the results, curcumin inhibits the expression and secretion of leptin and it could probably be used as a drug candidate for the breast cancer therapy through the leptin targeting in the future.

## Introduction

Breast cancer is one of most commonly diagnosed types of cancer among women in 2012 and expected to account for 29% (226,870) of all new cancer cases among women [1]. Many factors are involved in the breast carcinogenesis, including adipocytokines like leptin [2]. Leptin, a 167 amino acids hormone with a molecular mass of 16 kDa, is mainly secreted from adipose tissue [3]. It has central roles in the control of satiety, energy expenditure, food intake, many reproductive processes [4], affecting the metabolic and hematopoietic systems [5]. Beside the synthesis by adipose tissue as the main source [6], there have been identified other sources of leptin in the body including testicles [7], ovaries [6], placenta [8], cartilage and bone cells [9], skeletal muscle [10] and stomach [11]. Furthermore, the mitogenic, transforming or migration-induced properties of leptin have been revealed in many different cell types such as smooth muscle cells [12], normal and neoplastic colon cells [13, 14]; and also normal and malignant mammary epithelial cells [15, 16]. It has been shown that leptin induces growth and transformation in T47D breast cancer cells unlike normal breast epithelial cells [17]. Leptin acts through binding to its receptor known leptin receptor (ObR) located in the target cell membrane [18]. Significantly higher levels of both leptin and ObR expression have been found in cancer tissue relative to non-cancer epithelium [19]. Also, numerous breast cancer cell lines such as MCF-7 and T47D could express leptin and ObR [16]. All these observations confirm that leptin can act not only by endocrine and (or) paracrine action on mammary tumor cells, but also via an autocrine pathway. Additionally, a significant positive correlation has been obtained between leptin and ObR expressions with breast cancer tissue [20]. Therefore, this paracrine-autocrine leptin axis could become a target for leptin-inhibiting drugs in cancer treatment and prevention.

Results of a study showed that the ratio of ERα to ERβ, in human adipose tissue, was significantly correlated with the level of serum leptin in vivo [21]. Thus, ERα and ERβ maybe have different roles in the regulation of leptin expression.

Curcumin (diferuloylmethane), a derivative of turmeric is one of the most commonly used and highly researched phytochemicals. It exhibits promising pharmacological activities and has demonstrated beneficial effects in terms of cancer cell proliferation, growth, survival, apoptosis, migration, invasion, angiogenesis, and metastasis [22]. Considering important roles of leptin in the breast cancer biology, in this study we investigated the possible variations in the leptin secretion and expression as well as expression of ERs in the T47D breast cancer cell line after its treatment with pure curcumin.

## Methods

Our study has been conducted on cell lines and it is compatible with Helsinki ethical codes, and it has been approved by ethics committee of our institute.

### Chemicals and reagents

Curcumin (Sigma, Germany), MTT (Sigma, Germany), Leptin ELISA kit (Labor diagnostika nord gmbh & co. kg, Germany), Fetal bovine serum (Gibco, USA), Phenol-red free RPMI 1640 with L-glutamine (Gibco, USA), T47D cells (Pasteur Institute of Iran), Sodium bicarbonate (Merck, Germany), Penicillin (SERVA, Germany), Streptomycin (Merck, Germany), Amphotericin B (Merck, Germany), TRIZOL Reagent (Invitrogen, USA), First-Strand Synthesis kit (Fermentas, USA), Syber Green-I reagent (Fermentas, USA).

### Cell culture

T47D cells were cultured in RPMI1640 (with glutamine) supplemented with 10% FBS, penicillin, streptomycin and amphotericin B and incubated at 37°C in a humidified atmosphere containing 5% Co2.

### MTT assay and cell treatment

The cytotoxic effect of curcumin on T47D cells was studied by 24 and 48 MTT assays. Briefly, 2500 cell/well were cultivated in a 96 well culture plate. After 24 h incubation in 37°C cells were treated with different concentrations of curcumin (0 to 100 μM) for 24 and 48 h in the quadruplicate manner. Then, medium of all wells were removed carefully and 50 μl 2 mg/ml MTT was added to each well and incubated for 4.5 h, followed by addition of 200 μl DMSO. Thereafter, Sorensens’ glycine buffer was added and absorbance of each well was read at 570 nm. For data analysis, mean OD of each well was calculated. Then, percent of cells viability was calculated according to this formula: percent of cells viability = mean OD of test wells/mean OD of control wells × 100. Finally, a graph was plotted using SPSS 16.0 and IC50 of curcumin on T47D was determined on graph [23].

For studying the inhibitory effect of curcumin on leptin, ERα and ERβ expression as well as leptin secretion, 1×10^5^ cells/wells were treated in a 6-well plate with different concentrations of curcumin (0, 10, 20, 30 and 40 μM) for 24-48 h. A control group containing 0.1% DMSO without curcumin served as vehicle control.

### Isolation of total RNA and cDNA synthesis

Total RNA was extracted from attached cells using TRIZOL Reagent according to the manufacturer’s instructions. The concentration of prepared RNA was measured using a NanoDrop spectrophotometer (Termoscientific, USA) and its integrity was confirmed by electrophoresis on 1.5% agarose gel containing 1% formaldehyde. After RNA preparation, cDNA was synthesized using the First-Strand Synthesis kit according to the manufacturer’s instructions. The synthesized cDNA was immediately used in a real-time PCR or stored at −70˚C for later use.

### Real-time PCR

The real-time PCR was used for measurement of leptin, ERα, and ERβ expression levels in the control and treated cells. β-actin gene expression was used as the internal control. The real-time PCR reaction was done using the Syber Green-I reagent in the Rotor Gene 6000 system (Corbett research, Australia) according to the manufacturer’s instructions in a triplicate manner. The amplification conditions were as follows: leptin (2 min at 95°C and a two-step cycle of 95°C for 15 s and 60°C for 40 s for 40 cycles), ERα (5 min at 95°C and a two-step cycle of 95°C for 30 s and 57°C for 40 s for 40 cycles), and ERβ (5 min at 95°C and a two-step cycle of 95°C for 15 s and 64°C for 40 s for 40 cycles). Sequences of used primers were shown in Table 1. Changes in leptin, ERα, and ERβ expression levels between the control and treated T47D cells were calculated by the 2^–ΔΔCT^ method.

**Table 1:** **Primers used for real-time PCR amplifications**

Primer	Primer length	Sequence (5′ to 3′)	Product size (bp)
Leptin forward	22	CACCAAAACCCTCATCAAGACA	80
Leptin reverse	24	CTTTCTGTTTGGAGGAGACTGACT	
ERα forward	20	GCCAGCAGGTGCCCTACTAC	132
ERα reverse	23	TGGTACTGGCCAATCTTTCTCTG	
ERβ forward	19	AAGAGCTGCCAGGCCTGCC	268
ERβ reverse	21	GCGCACTGGGGCGGCTGATCA	
Β-actin forward	20	TGGACTTCGAGCAAGAGATG	137
Β-actin reverse	20	GAAGGAAGGCTGGAAGAGTG	

### Measurement of the secreted leptin

For analysis of possible effect of curcumin on amount of secreted leptin in the treated cells compared with the control cells, leptin concentration was measured in the supernatant media of cells using a human leptin ELISA kit according to the manufacturer’s instructions.

### Statistical analysis

Statistical analysis was performed with SPSS 18.0 software. Data are expressed as mean ± standard deviation. All experiments were performed in triplicate. The differences in expression levels of leptin, ERα and ERβ as well as quantity of secreted leptin between the control and treated cells were analyzed by one-way ANOVA, followed by Dunett’s multiple comparison tests. A p-value <0.05 was considered as significant.

## Results

### MTT assay

**Figure 1:**
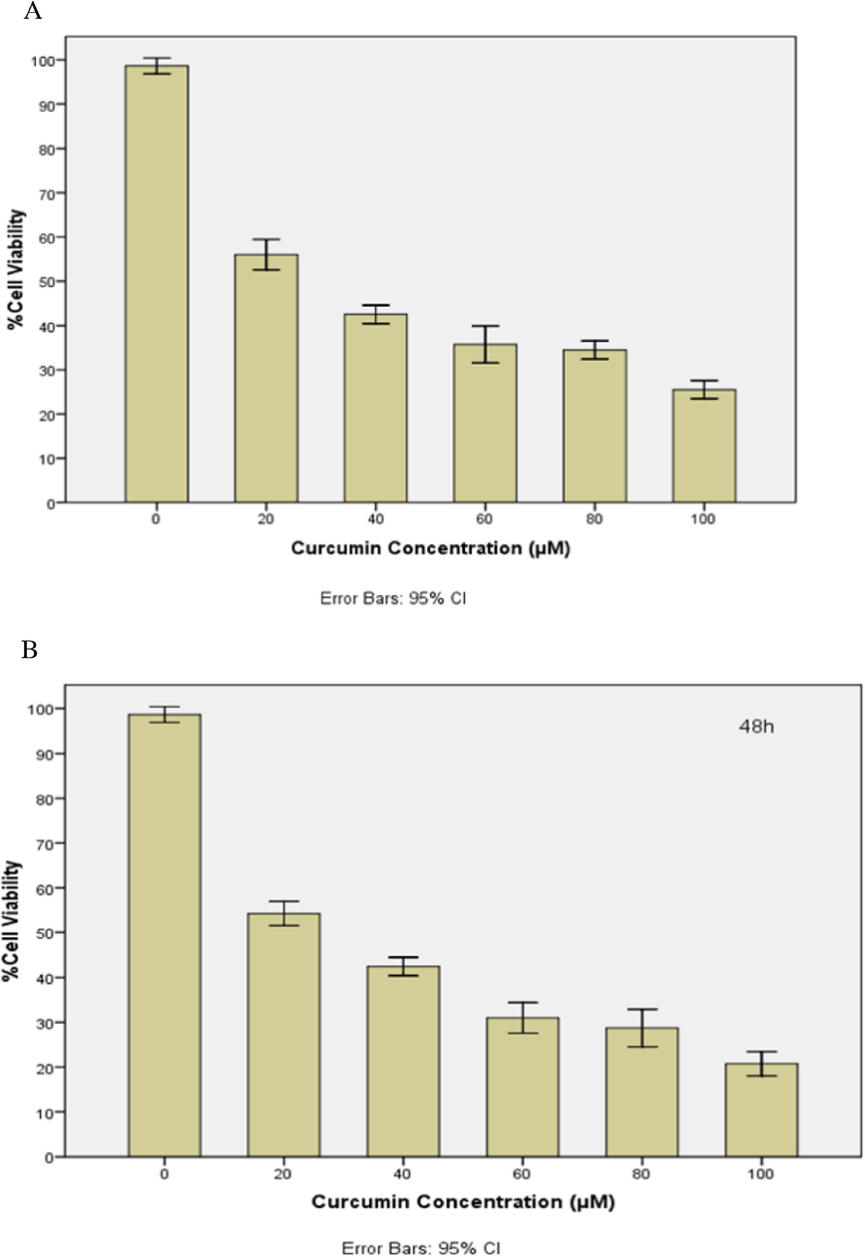
**The cytotoxic effect of curcumin on T47D cells during 24 h (A) and 48 h MTT assays.**

Data analysis of cytotoxicity assay showed that IC50 of curcumin on T47D breast cancer cell line was 28 and 24 μM for 24 and 48 h MTT assays, respectively (Figure 1). The obtained IC50s were dose-dependent.

### Quantitative real- time PCR

**Figure 2:**
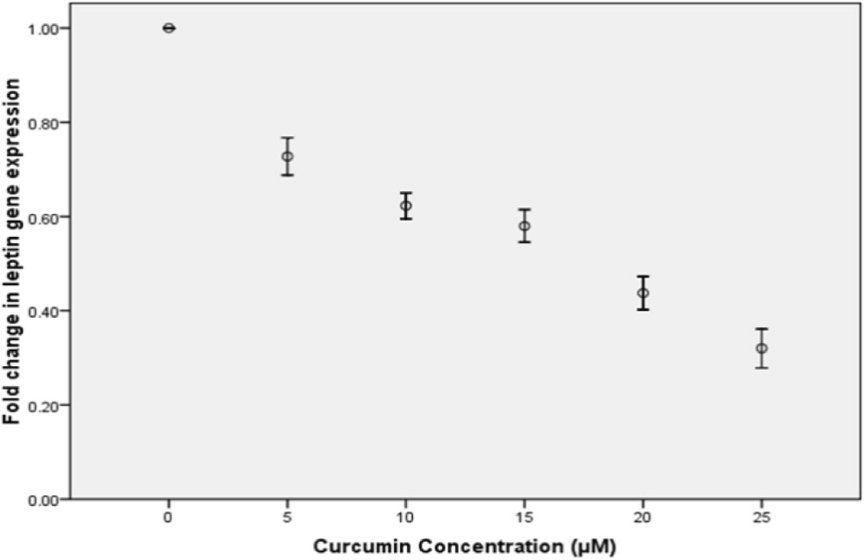
**Effect of curcumin on leptin expression levels in T47D cells.**

**Figure 3:**
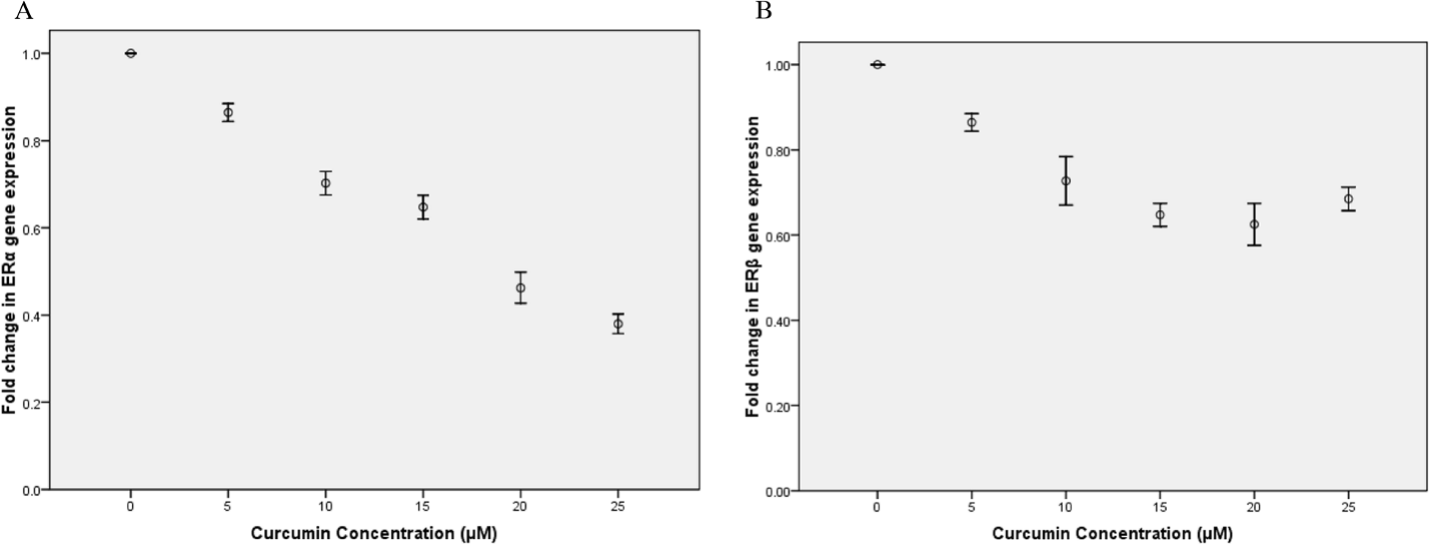
**Effect of curcumin on expression levels of ERα (A) and ERβ (B) genes in the T47D cells.** As the figure shows, there is a significant decrease in ERα gene expression levels with increasing in the curcumin concentration (p-value < 0.05), unlike ERβ gene expression levels.

Real-time PCR results showed a significant decrease in leptin expression in the treated cells compared to the control cells (p-value < 0.05) (Figure 2). We, also, measured the expression levels of ERα, and ERβ in the treated and control cells. Although, no significant difference was detected in ERβ expression levels between the treated and the control cells, a significant decrease was observed in the ERα mRNA level (p-value < 0.05) (Figure 3). Therefore, the ERα/ERβ expression ratio has been decreased in the treated cells compared to the control cells. There was no significant difference between the DMSO control and the DMSO-free control (the 0 μM concentration of curcumin).

Data analysis revealed a positive significant link between leptin gene expression and ERα gene expression level and no link between leptin and ERβ expression level in curcumin treated cells compared to the control cells. In addition, a positive significant correlation was found between mRNA level of leptin and ERα/ERβ expression ratio.

### Measurement of secreted leptin

**Figure 4:**
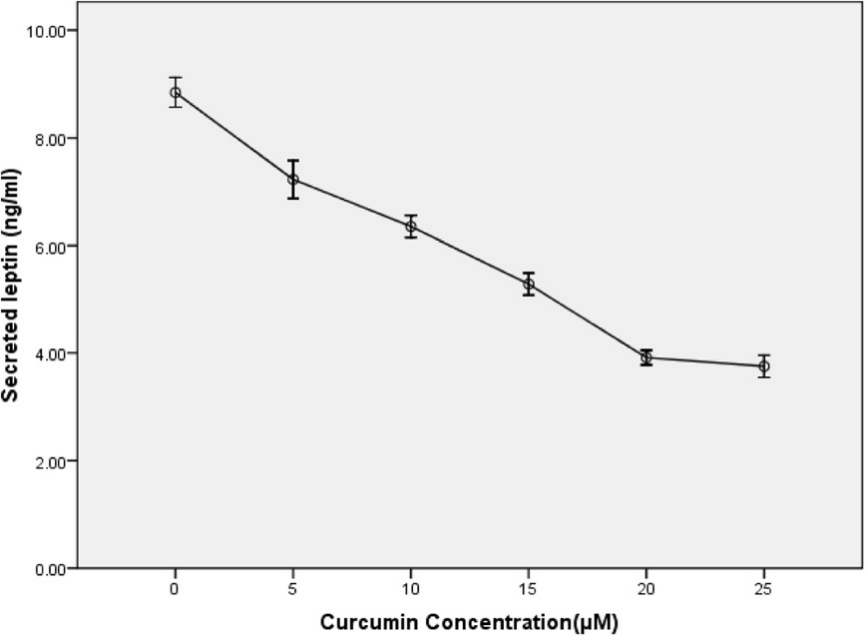
**Effect of curcumin on leptin secretion in T47D cells.**

Amounts of secreted leptin were evaluated using ELISA. A significant difference was found between the control and treated cells in term of secreted leptin (Figure 4). This finding was in accordance with inhibition of leptin gene expression by curcumin.

## Discussion

This study demonstrates that curcumin, most active constituent of turmeric , can inhibit leptin gene expression and secretion in T47D breast cancer cells and this decrease in leptin gene expression and secretion was positively associated with decrease of ERα gene expression. These results indicate that curcumin has a strong potential to interact with the expression of leptin gene, which has significant roles in carcinogenesis and proliferation of breast cancer cells [2]. Regarding to the critical role of leptin in breast carcinogenesis [24], there are many attempts to inhibit leptin function and secretion. Gonzalez et al. inhibited growth of murine mammary cancer cell and xenograft tumor model of human breast cancer cell lines by leptin peptide antagonist [25]. In addition, leptin analog mimicking its action [26] and anti-leptin receptor monoclonal antibody [27] are also another approaches for interfering with the leptin function. In the other studies, administrations of some compounds including b3-adrenoreceptor agonist, conjugated linoleic Acid, isoflavone, resveratrol and bitter melon lead to decreased secretion and lower levels of serum leptin [28]. The current work, however, aimed to direct inhibition of leptin expression and secretion in human T47D breast cancer cell line using curcumin. Due to significant anti-cancer effects of curcumin on various types of cancers such as gastrointestinal, genitourinary, gynecological, hematological, pulmonary, thymic, brain, breast, and bone [29], it can be used as a chemotherapeutic agent for breast cancer therapy.

The mechanisms by which curcumin exerts its anti-cancer effects are comprehensive and diverse, targeting many levels of regulation in the processes of cellular growth and apoptosis. Curcumin has an inhibitory effect on both NF-κB and AP-1 activation. curcumin has been shown to suppress the expression of a variety of NF-κB regulated gene products involved in carcinogenesis and tumor growth including cyclin D1, VEGF, COX-2, c-myc, Bcl-2, ICAM-1 and MMP-9 [30]. In addition, Curcumin has been shown to suppress the expression of cyclin D1 in many types of cancer including head and neck, colon, bladder, breast, cervical and pancreatic carcinomas [31]. Our results, regarding inhibition of leptin expression, confirm these findings, also demonstrating another role for curcumin effect that probably acts through estrogen receptors. It has been demonstrated that expression of leptin has positive correlation with expression of ERα and negative correlation with ERβ expression [32]. In addition, leptin promoter has response elements for both ERα and ERβ [33]; where, probably competition between ERα and ERβ in attachment to this place causes reduction of leptin gene transcription by lowered activity of the leptin promoter. Nejati-Koshki and et al. reported that silibinin can decrease leptin gene expression and secretion in T47D cells thought activation of ERβ gene expression [34]. In the current study, we showed that reduction of leptin expression by curcumin positively correlates with expression of ERα. Therefore, it seems that one of the possible mechanisms of curcumin inhibitory effect on leptin expression could be through ERs. However, many studies need to determine the exact molecular mechanism (s) involved in the reduction of leptin gene expression and secretion by curcumin [35–54].

## Conclusion

In summary, we demonstrated that curcumin could inhibit expression and secretion of leptin in T47D breast cancer cells. Regarding to the significant roles of leptin in breast carcinogenesis, its inhibition could be considered as a novel strategy for treatment of breast cancer in the future.

## References

[1] Siegel R, Naishadham D, Jemal A (2012). Cancer statistics, 2012. CA Cancer J Clin.

[2] Vona-Davis L, Rose DP (2007). Adipokines as endocrine, paracrine, and autocrine factors in breast cancer risk and progression. Endocr Relat Cancer.

[3] Zhang Y, Proenca R, Maffei M, Barone M, Leopold L, Friedman JM (1994). Positional cloning of the mouse obese gene and its human homologue. Nature.

[4] Collins S, Kuhn CM, Petro AE, Swick AG, Chrunyk BA, Surwit RS (1996). Role of leptin in fat regulation. Nature.

[5] Fantuzzi G, Faggioni R (2000). Leptin in the regulation of immunity, inflammation, and hematopoiesis. J Leukoc Biol.

[6] Löffler S, Aust G, Köhler U, Spanel-Borowski K (2001). Evidence of leptin expression in normal and polycystic human ovaries. Mol Hum Reprod.

[7] Soyupek S, Armağan A, Serel TA, Hoşcan MB, Perk H, Karaöz E, Candir O (2005). Leptin expression in the testicular tissue of fertile and infertile men. Arch Androl.

[8] Masuzaki H, Ogawa Y, Sagawa N, Hosoda K, Matsumoto T, Mise H, Nishimura H, Yoshimasa Y, Tanaka I, Mori T, Nakao K (1997). Nonadipose tissue production of leptin: leptin as a novel placenta-derived hormone in humans. Nat Med.

[9] Morroni M, De Matteis R, Palumbo C, Ferretti M, Villa I, Rubinacci A, Cinti S, Marotti G (2004). In vivo leptin expression in cartilage and bone cells of growing rats and adult humans. J Anat.

[10] Solberg R, Aas V, Thoresen GH, Kase ET, Drevon CA, Rustan AC, Reseland JE (2005). Leptin expression in human primary skeletal muscle cells is reduced during differentiation. J Cell Biochem.

[11] Mix H, Widjaja A, Jandl O, Cornberg M, Kaul A, Göke M, Beil W, Kuske M, Brabant G, Manns MP, Wagner S (2000). Expression of leptin and leptin receptor isoforms in the human stomach. Gut.

[12] Oda A, Taniguchi T, Yokoyama M (2001). Leptin stimulates rat aortic smooth muscle cell proliferation and migration. Kobe J Med Sci.

[13] Hardwick JC, Van Den Brink GR, Offerhaus GJ, Van Deventer SJ, Peppelenbosch MP (2001). Leptin is a growth factor for colonic epithelial cells. Gastroenterology.

[14] Liu Z, Uesaka T, Watanabe H, Kato N (2001). High fat diet enhances colonic cell proliferation and carcinogenesis in rats by elevating serum leptin. Int J Oncol.

[15] Dieudonne MN, Machinal-Quelin F, Serazin-Leroy V, Leneveu MC, Pecquery R, Giudicelli Y (2002). Leptin mediates a proliferative response in human MCF7 breast cancer cells. Biochem Biophys Res Commun.

[16] Laud K, Gourdou I, Pessemesse L, Peyrat JP, Djiane J (2002). Identification of leptin receptors in human breast cancer: functional activity in the T47-D breast cancer cell line. Mol Cell Endocrinol.

[17] Hu X, Juneja SC, Maihle NJ, Cleary MP (2002). Leptin—a growth factor in normal and malignant breast cells and for normal mammary gland development. J Natl Cancer Inst.

[18] Tartaglia LA (1997). The leptin receptor. J Biol Chem.

[19] Ishikawa M, Kitayama J, Nagawa H (2004). Enhanced expression of leptin and leptin receptor (OB-R) in human breast cancer. Clin Cancer Res.

[20] Koda M, Sulkowska M, Kanczuga-Koda L, Jarzabek K, Sulkowski S (2007). Expression of leptin and its receptor in female breast cancer in relation with selected apoptotic markers. Folia Histochem Cytobiol.

[21] Shin JH, Hur JY, Seo HS, Jeong YA, Lee JK, Oh MJ, Kim T, Saw HS, Kim SH (2007). The ratio of estrogen receptor alpha to estrogen receptor beta in adipose tissue is associated with leptin production and obesity. Steroids.

[22] Shehzad A, Wahid F, Lee YS (2010). Curcumin in cancer chemoprevention: molecular targets, pharmacokinetics, bioavailability, and clinical trials. Arch Pharm (Weinheim).

[23] Pourhassan M, Zarghami N, Rahmati M, Alibakhshi A, Ranjbari R (2010). The inhibitory effect of Curcuma longa extract on telomerase activity in A549 lung cancer cell line. Afr J Biotechnol.

[24] Garofalo C, Surmacz E (2006). Leptin and cancer. J Cell Physiol.

[25] Gonzalez RR, Watters A, Xu Y, Singh UP, Mann DR, Rueda BR, Penichet ML (2009). Leptin-signaling inhibition results in efficient anti-tumor activity in estrogen receptor positive or negative breast cancer. Breast Cancer Res.

[26] Peters JH, Simasko SM, Ritter RC (2007). Leptin analog antagonizes leptin effects on food intake and body weight but mimics leptin-induced vagal afferent activation. Endocrinology.

[27] Fazeli M, Zarkesh-Esfahani H, Wu Z, Maamra M, Bidlingmaier M, Pockley AG, Watson P, Matarese G, Strasburger CJ, Ross RJ (2006). Identification of a monoclonal antibody against the leptin receptor that acts as an antagonist and blocks human monocyte and T cell activation. J Immunol Methods.

[28] Ray A, Cleary MP (2010). Leptin as a potential therapeutic target for breast cancer prevention and treatment. Expert Opin Ther Targets.

[29] Shehzad A, Lee J, Lee YS (2013). Curcumin in various cancers. Biofactors.

[30] Kunnumakkara AB, Diagaradjane P, Anand P, Harikumar KB, Deorukhkar A, Gelovani J, Guha S, Krishnan S, Aggarwal BB (2009). Curcumin sensitizes human colorectal cancer to capecitabine by modulation of cyclin D1, COX-2, MMP-9, VEGF and CXCR4 expression in an orthotopic mouse model. Int J Cancer.

[31] Singh RP, Sharma G, Dhanalakshmi S, Agarwal C, Agarwal R (2009). Suppression of advanced human prostate tumor growth in athymic mice by silibinin feeding is associated with reduced cell proliferation, increased apoptosis, and inhibition of angiogenesis. Mol Cancer Ther.

[32] Yi KW, Shin JH, Seo HS, Lee JK, Oh MJ, Kim T, Saw HS, Kim SH, Hur JY (2008). Role of estrogen receptor-alpha and -beta in regulating leptin expression in 3 T3-L1 adipocytes. Obesity (Silver Spring).

[33] O’Neil JS, Burow ME, Green AE, McLachlan JA, Henson MC (2001). Effects of estrogen on leptin gene promoter activation in MCF-7 breast cancer and JEG-3 choriocarcinoma cells: selective regulation via estrogen receptors alpha and beta. Mol Cell Endocrinol.

[34] Nejati-Koshki K, Zarghami N, Pourhassan-Moghaddam M, Rahmati-Yamchi M, Mollazade M, Nasiri M, Esfahlan RJ, Barkhordari A, Tayefi-Nasrabadi H (2012). Inhibition of leptin gene expression and secretion by silibinin: possible role of estrogen receptors. Cytotechnology.

[35] Akbarzadeh A, Mikaeili H, Asgari D, Zarghami N, Mohammad R, Davaran S (2012). Preparation and in-vitro evaluation of doxorubicin-loaded Fe_3_O_4_ magnetic nanoparticles modified with biocompatible copolymers. Int J Nanomedicine.

[36] Akbarzadeh A, Zarghami N, Mikaeili H, Asgari D, Amir Mohammad G, Khaksar Khiabani H, Soodabeh D (2012). Synthesis, characterization and in vitro evaluation of novel polymer-coated magnetic nanoparticles for controlled delivery of doxorubicin. Int J Nanotechnol Sci Environ.

[37] Akbarzadeh A, Samiei M, Joo SW, Anzaby M, Hanifehpour Y, Nasrabadi HT, Davaran S (2012). Synthesis, characterization and in vitro studies of doxorubicin-loaded magnetic nanoparticles grafted to smart copolymers on A549 lung cancer cell line. J Nanobiotechnol.

[38] Zohreh E, Nosratollah Z, Manoutchehr K, Soumaye A, Abolfazl A, Mohammad R, Zohreh Mohammad T, Kazem N-K (2013). Inhibition of hTERT Gene Expression by Silibinin-Loaded PLGA-PEG-Fe3O4 in T47D Breast Cancer Cell Line. BioImpacts.

[39] Davaran S, Alimirzalu S, Nejati-Koshki K, Hamid Tayefi N, Abolfazl A, Amir Ahmad K, Mojtaba A, Somayeh A (2014). Synthesis and study of physicochemical characteristics of Fe3O4 magnetic nanocomposites based on poly (Nisopropylacrylamide) for anti-cancer drugs delivery. Asian Pac J Cancer Prev.

[40] Rogaie R-S, Nosratollah Z, Abolfazl B, Akram E, Abolfazl A, Mustafa R-T (2013). Studies of the relationship between structure and antioxidant activity in interesting systems, including tyrosol, hydroxytyrosol derivatives indicated by quantum chemical calculations. Soft.

[41] Nejati-Koshki K, Akbarzadeh A, pourhasan-Moghadam M, woo joo S (2013). Inhibition of leptin and leptin receptor gene expression by silibinin- curcumin combination. Asian Pac J Cancer Prev.

[42] Ghasemali S, Nejati-Koshki K, Akbarzadeh A, Tafsiri E, Zarghami N, Rahmati-Yamchi M, Alizadeh E, Barkhordari A, Tozihi M, Kordi S (2013). Study of inhibitory effect of β-Cyclodextrin-HelenalinComplex on HTERT Gene Expression in T47D breast cancer cell line by Real TimeQuantitative PCR (q-PCR). Asian Pac J Cancer Prev.

[43] Mollazade M, Nejati-Koshki K, Abolfazl A, Younes H, Zarghami N, Sang Woo J (2013). PAMAM Dendrimers arugment inhibitory effect of curcumin on cancer cell proliferation: possible inhibition of telomerase. Asian Pac J Cancer Prev.

[44] Davaran S, Rezaei A, Alimohammadi S, Amir Ahmad K, Kazem N-K, Hamid Tayefi N, Abolfazl A (2014). Synthesis and Physicochemical Characterization of Biodegradable star-shaped poly lactide-co-glycolide– β -cyclodextrin copolymer Nanoparticles Containing Albumin. Adv Nanoparticles.

[45] Davaran S, Abolfazl Akbarzadeh1, Kazem N-K, Somayeh A, Mahmoud Farajpour G, Mahsa Mahmoudi S, Akbar R, Amir Ahmad K (2013). In vitro studies of NIPAAM-MAA-VP copolymer-coated magnetic nanoparticles for controlled anticancer drug release. J Encapsulation Adsorption Sci.

[46] Nasiri M, Zarghami N, Koshki KN, Mollazadeh M, Moghaddam MP, Yamchi MR, Esfahlan RJ, Barkhordari A, Alibakhshi A (2013). Curcumin and silibinin inhibit telomerase expression in T47D human breast cancer cells. Asian Pac J Cancer Prev.

[47] Abolfazl A, Samiei M, Soodabeh D (2012). Magnetic nanoparticles: preparation, physical properties and applications in biomedicine. Nanoscale Res Lett.

[48] Alireza V, Haleh M, Mohammad S, Samad Mussa F, Nosratollah Z, Mohammad K, Abolfazl A, Soodabeh D (2012). Quantum dots: synthesis, bioapplications, and toxicity. Nanoscale Res Lett.

[49] Abolfazl A, Rogaie R-S, Soodabeh D, Sang Woo J, Nosratollah Z, Younes H, Mohammad S, Mohammad K, Kazem N-K (2013). Liposome: classification, preparation, and applications. Nanoscale Res Lett.

[50] Mohammad P-M, Mohammad R-Y, Abolfazl A, Hadis D, Kazem N-K, Younes H, Sang Woo J (2013). Protein detection through different platforms of immuno-loop-mediated isothermal amplification. Nanoscale Res Lett.

[51] Ebrahimnezhad Z, Zarghami N, Keyhani M, Amirsaadat S, Akbarzadeh A, Rahmati M, Mohammad Taheri Z, Nejati-Koshki K (2013). Inhibition of hTERT Gene Expression by Silibinin-Loaded PLGA-PEG-Fe3O4 in T47D Breast Cancer Cell Line. Bioimpacts.

[52] Pourhassan-Moghaddam M, Rahmati-Yamchi M, Akbarzadeh A, Daraee H, Nejati-Koshki K, Hanifehpour Y, Joo SW (2013). Protein detection through different platforms of immuno-loop-mediated isothermal amplification. Nanoscale Res Lett.

[53] Esfahlan RJ, Zarghami N, Esfahlan AJ, Mollazadeh M, Nejati K, Nasiri M (2011). Basic of DNA biosensors and cancer diagnosis. The Possible Impact of Obesity on Androgen, Progesterone and Estrogen Receptors (ERα and ERβ) Gene Expression in Breast Cancer Patients. Breast Cancer (Auckl).

[54] Mirakabad FST, Akbarzadeh A, Zarghami N, Zeighamian V, Rahimzadeh A, Alimohammadi S (2014). PLGA-Cased nanoparticles as cancer drug delivery systems. APJCP Asian Pac J Cancer Prev.

